# Prevalence and clinical implications of bloodstream infections in intensive care patients with or without burn injury: a retrospective cohort study

**DOI:** 10.1007/s10096-024-04877-w

**Published:** 2024-06-26

**Authors:** Felix Bergmann, Anselm Jorda, Julia Sollner, Rebecca Sawodny, Kerstin Kammerer, Valerie List, Marlene Prager, Georg Gelbenegger, Katarina Kumpf, Heimo Lagler, Markus Zeitlinger, Christine Radtke

**Affiliations:** 1 Department of Plastic, Reconstructive and Aesthetic Surgery, Medical University of Vienna, Währinger Gürtel 18-20, Vienna, 1090 Austria; 2 Department of Clinical Pharmacology, Medical University of Vienna, Vienna, Austria; 3 Department of Pathology, Medical University of Vienna, Vienna, Austria; 4 Department of Plastic and Reconstructive Surgery, Hospital Wiener Neustadt, Wiener Neustadt, Austria; 5 IT Systems and Communications, Medical University of Vienna, Vienna, Austria; 6 Division of Infectious Diseases and Tropical Medicine, Department of Medicine I, Medical University of Vienna, Vienna, Austria

**Keywords:** Thermal injury, Antibiotics, ICU, Critical care, Sepsis, Bacteremia

## Abstract

**Purpose:**

Severe burn injuries are often accompanied by infections and associated with high morbidity and mortality. This study aimed to compare the prevalence and clinical impact of bacteremia between patients receiving intensive care with and without burns.

**Methods:**

This single-center retrospective cohort study at the University Hospital Vienna, Austria, analyzed blood cultures from intensive care unit (ICU) patients with and without burns (2012–2022) to assess the prevalence of bacteremia, the associated pathogen distribution and the 60-day all-cause mortality.

**Results:**

In 1170 ICU patients, 303 with burns and 867 without, the prevalence of bacteremia was similar among patients with at least one blood culture (31/157 [19.7%] versus 44/213 [20.7%], OR [95%CI] = 0.95 [0.57–1.57]). Burn patients exhibited a significantly higher frequency of microbiological sampling (51.5% versus 24.5%, *p* < 0.001), resulting in a higher overall prevalence of bacteremia (10.2% versus 5.1%, *p* = 0.002). 16.2% of all identified pathogens were multidrug-resistant (MDR). The 60-day all-cause mortality was higher in patients with MDR pathogens than in patients without bacteremia (41.7% versus 10.6%, *p* = 0.026).

**Conclusion:**

Bacteremia prevalence was similar in burn and non-burn patients, with high rates of multidrug-resistant Gram-negative pathogens. The 60-day all-cause mortality was significantly higher in patients with MDR pathogens than in patients without bacteremia.

## Introduction

According to the World Health Organization, burn injuries cause an estimated 180,000 fatalities per year worldwide, predominantly in low- and middle-income countries [1]. Despite a declining trend in mortality, particularly in high-income regions [2], burn wounds and associated infections remain a global challenge. Following severe thermal injury, inflammatory processes lead to a state of immunosuppression [3]. In addition, the compromised skin integrity promotes the invasion of various pathogens, which renders severely burned patients highly susceptible to inflammatory response syndrome or sepsis [3].

Several studies investigated the pathogen prevalence and distribution in patients receiving intensive care. However, the findings have been heterogeneous, showcasing varying results. In patients with severe thermal trauma, 4 to 28% developed bloodstream infections, which are associated with high morbidity and mortality [4–7]. While the causative microorganisms may differ between countries and hospitals, the pathogens of bloodstream infections of burn patients are typically Gram-negative [5, 7].

In contrast, previous studies have documented a lower prevalence of bacteremia in patients treated in the ICU without thermal trauma, with prevalence figures ranging from 2 to 11% [8–10]. While certain studies suggested a predominance of gram-positive pathogens like coagulase-negative Staphylococci, *Staphylococcus aureus*, or *Enterococcus spp.* [11–13], contrasting investigations have reported a higher prevalence of Gram-negative bacteria [14].

While both burn ICU patients and general ICU patients are at a high risk for bloodstream infections, burn ICU patients are often considered to be at a higher risk due to the combination of compromised skin barriers, prolonged ICU stays, and impaired immune responses. Although previous research projects have analyzed the incidence and underlying pathogen spectrum of bacteremia in burn patients and general ICU patients individually, a direct comparison between these two groups has yet to be conducted. Hence, this study presents a direct comparison of the prevalence and clinical impact of bacteremia in intensive care patients with burns versus those without burns.

## Materials and methods

### Study design and setting

This single-center, retrospective cohort study was conducted at the University Hospital of Vienna, Austria. The research adhered to ethical principles in line with the Declaration of Helsinki. The project received approval from the local Ethics Committee (Ethics Committee of the Medical University of Vienna) with the reference EC 2259/2021. The extraction of data from electronic medical records was automated and a manual review was conducted by two independent reviewers to address any implausible or missing data. The reporting of this observational study followed the STROBE (Strengthening the Reporting of Observational Studies in Epidemiology) recommendations [15].

### Study population

The burn intensive care unit (BICU) at the University Hospital of Vienna comprises 6 ICU beds and is designed as a specialized facility for the management of patients with severe burn or scald injuries. Nevertheless, contingent upon available capacities, the unit is open to admitting and managing patients requiring intensive care, irrespective of the presence of burn injuries. Non-burn patients include subjects with a wide spectrum of medical and surgical conditions.

We included patients who were hospitalized and admitted to the BICU with or without burns from 2012 to 2022. Key inclusion criteria were direct admission to the ICU (including patients with a length of stay [LOS] ≤ 24 h at the normal ward), male and female patients over 18 years of age, and a LOS ≥ 3 days at the BICU. Specific inclusion criteria for patients with burns or scalds were an affected total body surface area (TBSA) of ≥ 10% and a burn degree of ≥ IIa°.

### Microbiological definitions

Bacteremia was defined by a positive microbiological culture of relevant bacterial species in blood samples. Only samples from blood were considered in this analysis. We excluded fungal pathogens, staphylococci other than *S. aureus*, and commensal skin bacteria (i.e. *Cutibacterium* and *Corynebacterium* spp.), which are commonly the result from contamination.

### Outcome parameters

The primary outcome of this study was the frequency of positive microbiological cultures in blood samples from patients with versus without burns during the initial four weeks of admission. The primary outcome specifically included patients who underwent blood culture testing at least once, since a higher testing frequency may result in a higher positivity rate. Frequencies were described overall and per week 1 to 4. In addition, only those patients who were still hospitalized in the respective week were considered in the positivity rates per week. Secondary outcomes included the testing frequency in both groups, the overall frequency of positive microbiological blood cultures in both groups (i.e. regardless of whether subjects underwent at least one test), the distribution and prevalence of pathogens, the prevalence of multidrug-resistant (MDR) pathogens and the 60-day all-cause mortality. MDR definitions followed those proposed by the European Centre for Disease Prevention and Control [16].

### Statistical analysis

Numerical baseline characteristics were presented descriptively using mean ± standard deviation (SD) or median with interquartile range (Q1 - Q3), depending on the data distribution. Frequencies are reported as numbers with percentages (%). Independent t-test (for age, weight, height, and body mass index [BMI]) or Chi-square test (for sex and chronic diseases) was employed for between-group comparisons. Analyses of the primary and secondary endpoints were performed using Fishers-exact test. Odds ratios (OR) of the primary and secondary endpoints were covariate-adjusted using a generalized linear model. Crude and covariate-adjusted odds ratios were reported throughout. Covariates were chosen based on their potential interaction with the occurrence of infections. These covariates included age, sex, BMI, Sequential Organ Failure Assessment (SOFA) score at baseline, antibiotic use at baseline and comorbidities such as cardiovascular disease, psychiatric conditions, diabetes, neoplasms, and hypertension. Corresponding confidence intervals were computed using the Baptista-Pike method [17]. Hazard ratios with a 95% confidence interval (95% CI) for 60-day all-cause mortality were calculated using the Mantel-Haenszel method. P-values of the survival analyses were calculated with the log-rank test. Descriptive reporting covered pathogen prevalence and distribution and MDR prevalence. The statistical analysis and visualization were conducted using R (Version 2021.09.2) and GraphPad Prism 9.3.1, respectively.

## Results

### Study population

**Fig. 1:**
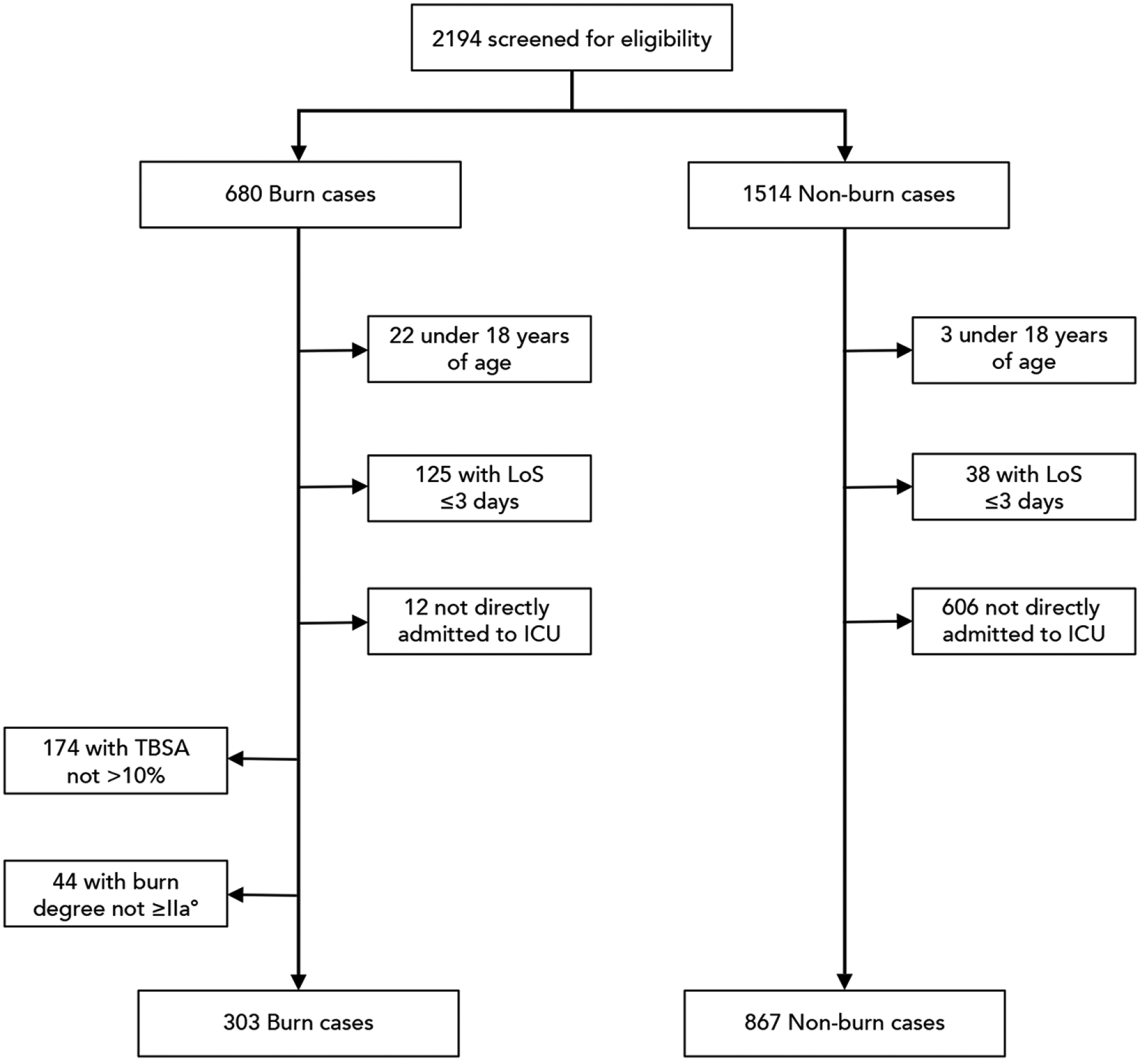
Flow chart of the study population

From 2012 to 2022, a total of 2194 patients were admitted to the investigated intensive care unit and automatically extracted (Fig. 1). We excluded 25 patients aged under 18 years, 163 patients with a length of stay of three days or less, and 618 patients because they were not directly admitted to the ICU. In the burn group, 174 patients and 44 patients were excluded because of a low TBSA and burn degrees, respectively. The final analysis included a total of 1170 eligible patients, including 303 burn cases and 867 non-burn cases (Fig. 1). Table 1 shows the patient characteristics of the study population. The burn group had a higher proportion of male subjects (73.3% versus 57.7%), was considerably younger (47 [34–65] versus 61 [48–72] years) and displayed a significantly longer length of ICU stay (26.5 [14.9–47.2] versus 13.3 [9.1–25.6] days) than patients in the non-burn group. In patients who received mechanical ventilation, the median duration of mechanical ventilation was significanntly longer in patients with burn trauma versus patients without (23 [11–47] versus 9 [3–29]). Both groups had a similar duration of stay of arterial-, central venous-, and urinary catheter before removal or replacement. The most common reasons for ICU admission in the non-burn group were cardiovascular disease (397 of 867 [45.8%]), neoplasm (220 of 867 [25.4%]) and metabolic disease (190 of 867 [21.9%]). Concerning comorbidities, in the burn group, there was a significantly lower frequency of cardiovascular diseases (38 of 303 [12.5%] versus 178 of 867 [20.5%], *p* = 0.003), hematological diseases (6 [2.0%] versus 70 [8.1%], *p* < 0.001) and neoplasms (3 [1%] versus 189 [21.8%], *p* < 0.001) than in the non-burn group. In contrast, the prevalence of psychiatric disorders was more frequent in the burn than in the non-burn group (33 [10.9%], versus 37 [4.3%], *p* < 0.001).

**Table 1:** Baseline characteristics of the study cohorts

	Overall (*n* = 1170)	Burn (*n* = 303)	Non-burn (*n* = 867)	*p*-value
Demographic characteristics				
Male sex, n (%)	722 (61.7)	222 (73.3)	500 (57.7)	< 0.001
Age (years), median (IQR)	58 (42–71)	47 (34–65)	61 (48–72)	< 0.001
BMI, mean (SD)	27.9 (15.2)	26.6 (5.3)	28.3 (17.0)	0.213
**Length of ICU stay (days)**, median (IQR)	16.2 (9.2–34.7)	26.5 (14.9–47.2)	13.3 (9.1–25.6)	< 0.001
**SOFA score at ICU admission**, median (IQR)	4 (3–7)	4 (1–7)	5 (3–7)	0.008
**Highest SOFA score during ICU stay**, median (IQR)	6 (4–9)	7.5 (4–10)	5 (3–8)	< 0.001
**Duration of mechanical ventilation_(days)**, median (IQR)	14 (4–37)	23 (11–47)	9 (3–29)	< 0.001
**Duration of the catheter before removal or replacement (days)**, median (IQR)				
Arterial catheter	5 (2–10)	4 (2–9)	5 (2–10)	0.0387
Central venous catheter	9 (3–14)	8 (3–14)	9 (4–13)	0.7451
Urinary catheter	6 (3–14)	5 (2–11)	6 (3–14)	0.0323
Reasons for ICU admission*, n (%)				
Burn	303 (25.9)	303 (100)	0 (0)	
Cardiovascular disease	397 (33.9)	0 (0)	397 (45.8)	
Neoplasm	220 (18.8)	0 (0)	220 (25.4)	
Metabolic disease	190 (16.2)	0 (0)	190 (21.9)	
Respiratory tract disease	151 (12.9)	0 (0)	151 (17.49	
Endocrine disease	57 (4.9)	0 (0)	57 (6.6)	
**Baseline comorbidities**				
Cardiovascular disease	216 (18.5)	38 (12.5)	178 (20.5)	0.003
Dermatological disease	18 (1.5)	1 (0.3)	17 (2.0)	0.086
Diabetes	54 (4.6)	9 (3.0)	45 (5.2)	0.154
Endocrine disease	26 (2.2)	5 (1.7)	21 (2.4)	0.577
Gastrointestinal disease	31 (2.6)	3 (1.0)	28 (3.2)	0.060
Haematologic disease	76 (6.5)	6 (2.0)	70 (8.1)	< 0.001
Hepatic disease	30 (2.6)	5 (1.7)	25 (2.9)	0.338
Hypertension	145 (12.4)	28 (9.2)	117 (13.5)	0.067
Neurological disease	57 (4.9)	8 (2.6)	49 (5.7)	0.052
Neoplasm	192 (16.4)	3 (1.0)	189 (21.8)	< 0.001
Psychiatric disorders	70 (6.0)	33 (10.9)	37 (4.3)	< 0.001
**Antibiotic use at admission**, n (%)	769 (65.7)	145 (47.9)	624 (72.0)	< 0.001
**TBSA (%)**, median (IQR)		24 (16–39)		
**III° burn**, n (%)		187 (61.7)		
**ABSI score**, median (IQR)		5 (4–7)		

* Listed are the most common reasons for ICU admission. Percentages do not sum to 100% because most patients had multiple reasons for ICU admission. Median days of ventilation refer only to patients who were mechanically ventilated. ABSI Abbreviated Burn Severity Index; BMI Body Mass Index; SOFA Sequential Organ Failure Assessment TBSA Total Body Surface Area affected by burn

Burn patients had a median (IQR) total body surface area (TBSA) of 24% (16–39) and 61.7% of patients had III° burned or scalded areas. The median (IQR) abbreviated burn severity index (ABSI) score was 5 (4–7).

Antibiotic use at ICU admission was significantly higher in the non-burn group than burn group; 624 (72%) of non-burn patients and 145 (47.9%) received antibiotics within three days of ICU admission. Table 2 provides an overview of antibiotic use at admission (i.e. within the first 3 days of ICU admission) in both groups. In non-burn patients, the most frequent antibiotics were Cephalosporines (2nd- or 3rd- generation) (used in 280 of 867 [32.2%] patients) and beta-lactams with beta-lactamase inhibitors (used in 207 [23.9%] patients). In the burn group, the most frequently used antibiotics were beta-lactams with beta-lactamase inhibitors (used in 138 of 303 [45.4%] patients) and macrolides (used in 15 [5%] patients).

**Table 2:** Antibiotic use within three days of ICU admission

Antibiotic, *n* (%)	Burn (*n* = 303)	Non-burn (*n* = 867)
Any antibiotic	145 (47.9)	624 (72.0)
Cephalosporine 2. /3. Gen	3 (1.1)	280 (32.2)
Beta-Lactam/BLI	138 (45.5)	207 (23.9)
Metronidazole	2 (0.7)	159 (18.3)
Cephalosporine 1. Gen	0 (0)	77 (8.9)
Clindamycin	3 (1.0)	66 (7.6)
Carbapenems	5 (1.7)	37 (4.3)
Fluoroquinolone	4 (1.3)	28 (3.2)
Fosfomycin	0 (0)	19 (2.2)
Linezolid	0 (0)	12 (1.4)
Macrolide	15 (5.0)	11 (1.3)
Penicillins	0 (0)	9 (1.0)
Tetracycline	0 (0)	8 (0.9)
Vancomycin	0 (0)	5 (0.6)
Cephalosporine 4. /5. Gen	0 (0)	3 (0.3)
Co-trimoxazole	0 (0)	3 (0.3)
Daptomycin	0 (0)	3 (0.3)
Rifampicin	0 (0)	2 (0.2)
Aminoglycosides	0 (0)	1 (0.1)

Gen generation; Beta-Lactam/BLI beta-lactam antibiotic and beta-lactamase inhibitor

### Prevalence of bloodstream infections

**Fig. 2:**
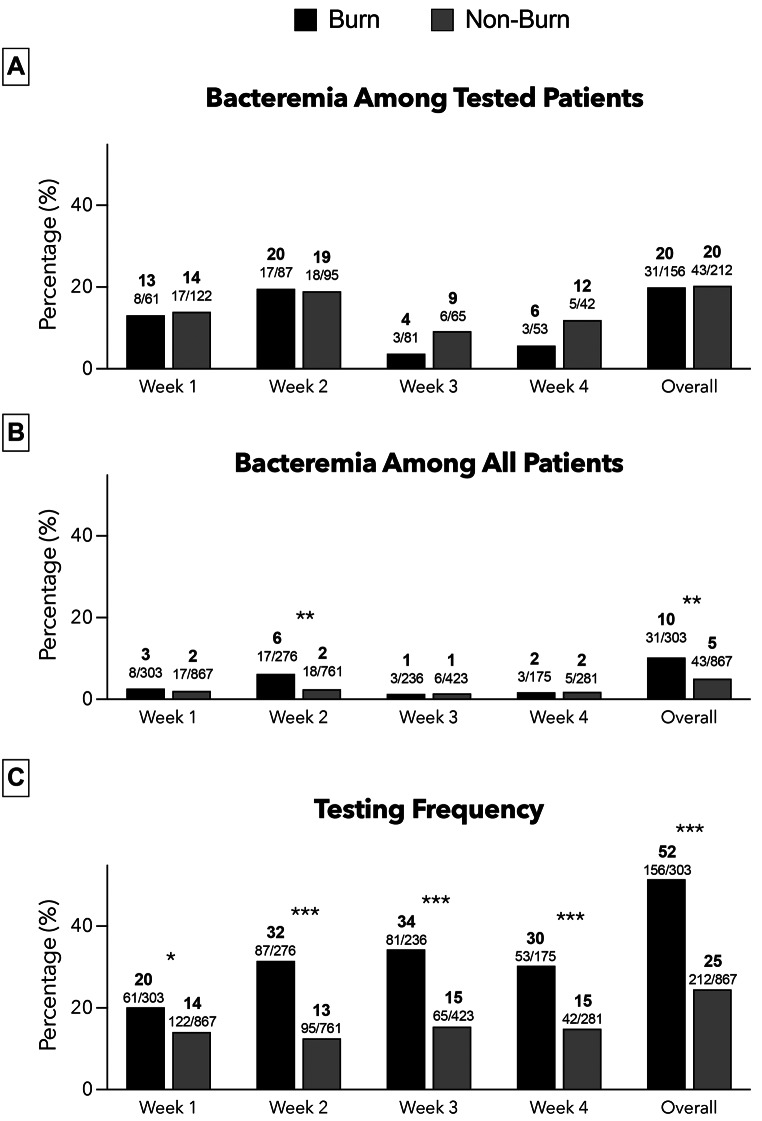
(**A**) Frequency of bacteremia in patients with at least one microbiological sample, (**B**) Frequency of bacteremia in all patients, (**C**) Microbiological testing frequency; Bold numbers represent frequencies in %; Regular numbers represent n/n; *, **, and *** refer to unadjusted p-values of ≤ 0.05, ≤ 0.01 and ≤ 0.001, respectively; Overall refers to the overall frequencies observed in weeks 1 to 4

Figure 2 provides an overview of the prevalence of positive microbiological cultures among patients with versus without burns during the initial four weeks of admission. In patients with at least one blood culture test, the prevalence of bacteremia (i.e., the primary endpoint) was similar between patients with versus without burns (31 of 157 [19.7%] versus 44 of 213 [20.7%], crude OR [95%CI] = 0.95 [0.57–1.57], adjusted OR [95%CI] = 1.07 [0.90–1.27]) (Fig. 2a). The frequency of bacteremia was almost identical between the two groups in the first (13% in burn group versus 14% in non-burn group) and second week (20% in burn group versus 19% in non-burn group). The frequency of bacteremia was numerically lower in burn than in non-burn patients in the third (4% versus 9%) and fourth week (6% versus 12%), without reaching statistical significance.

The overall prevalence of bacteremia, independent of testing, was higher in the burn group than in the non-burn group (31 of 303 [10.2%] versus 44 of 867 [5.1%], crude OR [95%CI] = 2.13 [1.33–3.41], crude OR [95%CI] = 1.08 [1.01–1.15]) (Fig. 2B). This difference was mainly driven by a higher frequency of bacteremia in the burn group than in the non-burn group during the second week (6% versus 2%).

In all four weeks, burn cases exhibited a significantly higher frequency of microbiological sampling compared with the non-burn cases (Fig. 2C). Any microbiological blood sampling was performed in 156 of 303 (51.5%) patients in the burn group and in 212 of 867 (24.5%) patients in the non-burn group (crude OR [95%CI] = 3.28 [2.50–4.31], (adjusted OR [95%CI] = 1.44 [1.27–1.62]). The frequency of blood culture sampling was constant from week 1 to 4 in the non-burn group (14%, 13%, 15%, and 15%, respectively). In the burn group, the frequency of blood culture sampling was lower in week 1 compared to week 2 to 4 (20%, 32%, 34%, 30%, respectively).

### Pathogen distribution

**Fig. 3:**
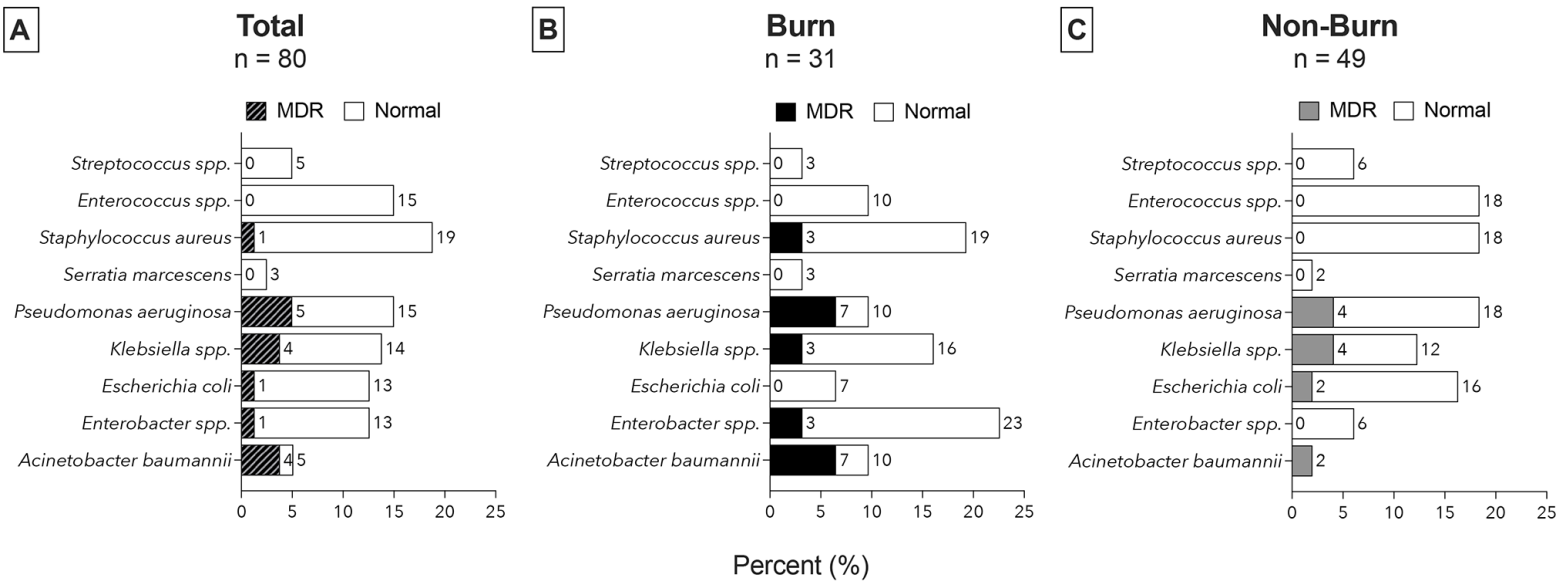
Relative frequencies (%) of bacterial species and prevalence of MDR pathogens in blood cultures in (**A**) total, (**B**) burn group and (**C**) non-burn group; Shaded areas represent the proportion of MDR strains of the respective pathogens; MDR Multi Drug Resistant

Figure 3 depicts the relative frequency of the identified pathogens and the proportion of MDR bacterial pathogens. Overall, 80 pathogens were identified in 74 patients, of which 31 (38.8%) were Gram-positive and 49 (61.3%) Gram-negative. Gram-positive pathogens accounted for 10 of 31 (32.3%) pathogens in the burn group and 21 of 49 (42.9%) pathogens in the non-burn group. Gram-negative pathogens accounted for 21 of 31 (67.7%) pathogens in the burn group and 28 of 49 (57.1%) pathogens in the non-burn group.

**Fig. 4:**
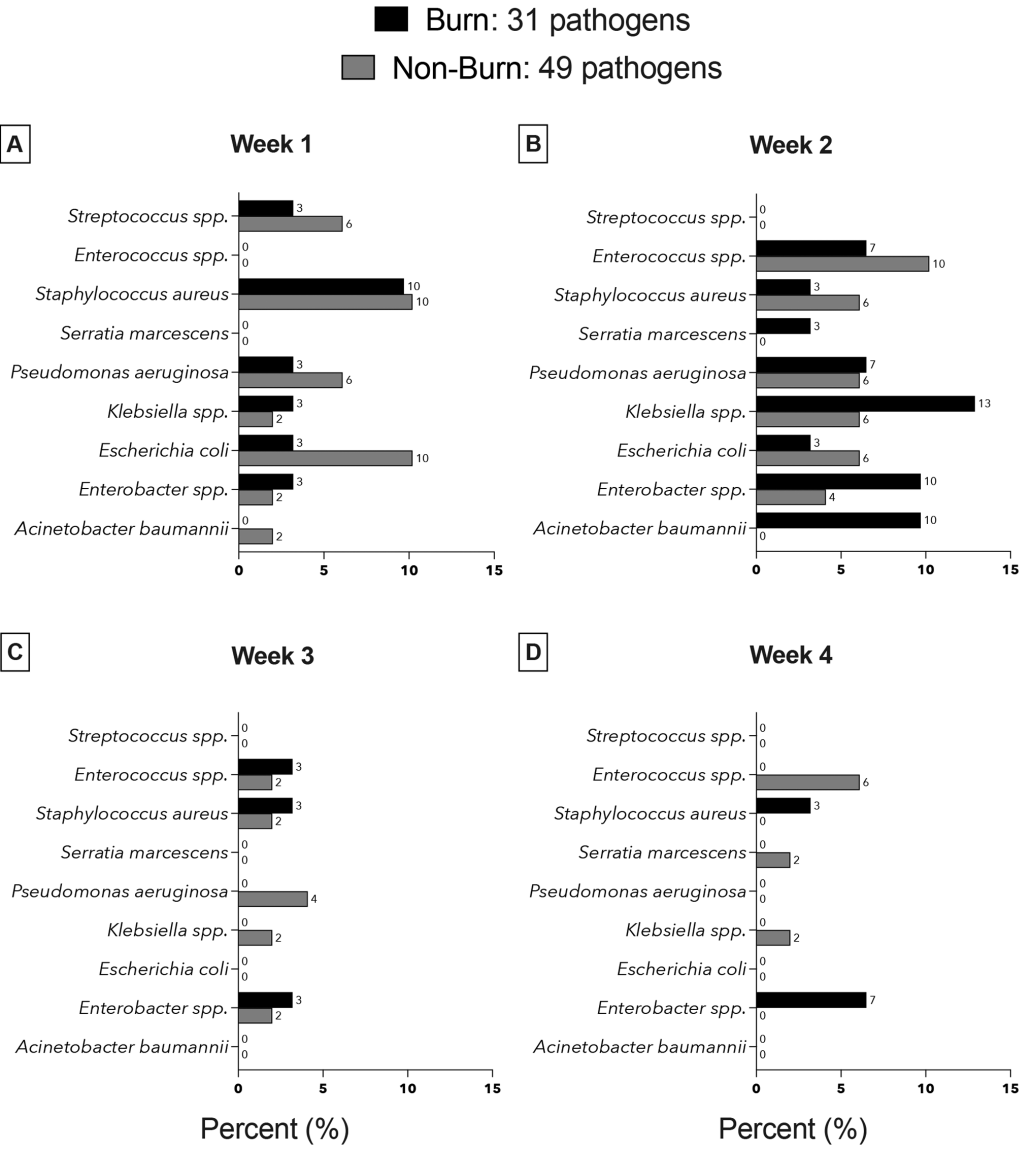
Relative frequency (%) of bacterial species in blood cultures of burn and non-burn patients in weeks 1 to 4 (**A**-**D**). Percentages refer to the total number of pathogens within each group

In the burn group, Enterobacter spp. (7 of 31 [22.6%]), *S. aureus* (6 of 31 [19.3%]) and *Klebsiella* spp. (5 of 31 [15.9%]) were the most frequent pathogens. In the non-burn group, *Enterococcus* spp., *S. aureus*, and *Pseudomonas aeruginosa* were the predominant pathogens (9 of 49 [18.4%] each). Figure 4 provides a temporal breakdown of pathogen distribution among patients with and without burns in the initial four weeks following ICU admission. The highest prevalence was observed in the first two weeks of admission. Multidrug resistance, predominantly observed in Gram-negative bacteria, was identified in 13 of 80 (16.2%) pathogens. Pathogens exhibiting MDR included *S. aureus, P. aeruginosa, Klebsiella pneumoniae, Escherichia coli*, Enterobacter spp., and *Acinetobacter baumannii.*

### Mortality

**Fig. 5:**
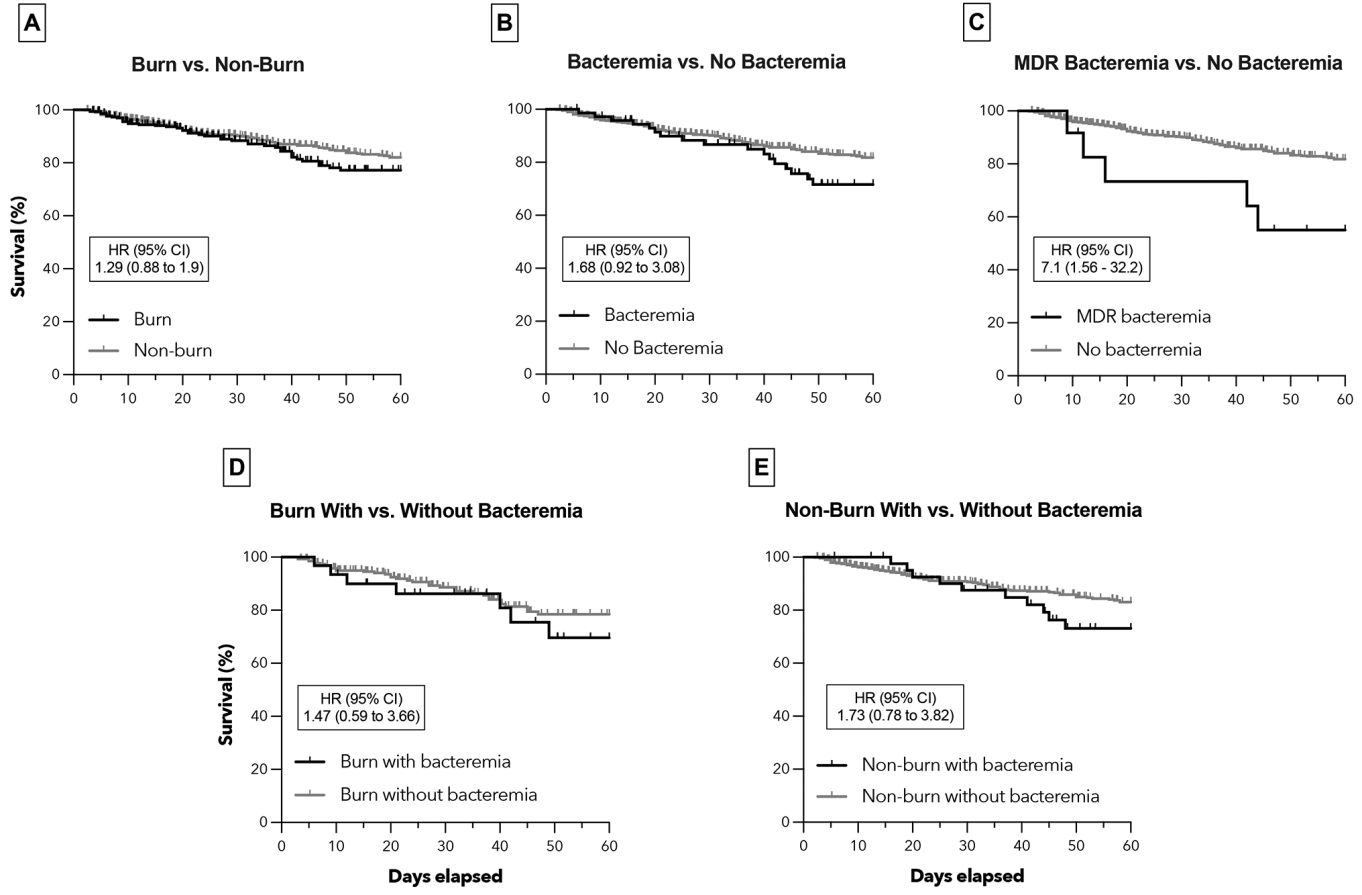
60-day all-cause mortality after ICU admission (**A**) Comparison of survival between the burn and non-burn group (**B**) Comparison of survival between patients with and without bacteremia (**C**) Comparison of survival between patients with bacteremia due to MDR bacteria and without bacteremia (**D**) Comparison of survival between burn cases with and without bacteremia (**E**) Comparison of survival between non-burn cases with and without bacteremia cases with and without bacteremia (**E**) Comparison of survival between non-burn cases with and without bacteremia

The 60-day all-cause mortality was similar between patients with versus without burns (43 of 303 [14.2%] versus 90 of 867 [10.4%], HR [95%CI] = 1.29 [0.88 − 0.53], *p* = 0.286) (Fig. 5A). Similarly, no statistically significant difference in mortality was observed in patients with versus without bacteremia (17 of 74 [23%] versus 116 of 1096 [10.6%], HR [95%CI] = 1.68 [0.92 − 0.32], *p* = 0.349) (Fig. 5B). Mortality was significantly higher in patients with a confirmed MDR bacterial pathogen than in patients without any positive blood culture (5 of 12 [41.7%] versus 116 of 1096 [10.6%], HR [95%CI] = 7.1 [1.56–32.24], *p* = 0.026) (Fig. 5C). There was no difference in mortality in burn patients with versus without bacteremia (7 of 31 [22.6%] versus 36 of 272 [13.2%], HR [95%CI] = 1.47 [0.59–0.68], *p* = 0.466) (Fig. 5D). Similarly, in patients without burns, there was no difference in mortality in patients with versus without bacteremia (10 of 43 [23.3%] versus 80 of 824 [9.7%], HR [95%CI] = 1.73 [0.78 − 0.26], *p* = 0.646) (Fig. 5E).

## Discussion

This is the first study to directly compare the prevalence and clinical implications of bacteremia between intensive care patients with versus without burns. In patients who were tested at least once, the frequency of bacteremia was similar between burn (19.7%) and non-burn patients (20.7%) admitted to the ICU. This finding contradicts the typical anticipation of a greater occurrence of bacteremia in patients with burn injuries. Notably, our study identified a significantly higher frequency of microbiological sampling in burn patients (51.5% tested at least once) than in non-burn patients (24.5%), a practice likely influenced by increased vigilance for infections in this demographic. Consequently, the frequency of bacteremia in all patients, independent of testing, was significantly higher in burn patients (10.2%) compared to non-burn patients (5.1%). These frequencies correlate more closely with the acknowledged vulnerability of burn patients to bloodstream infections resulting from compromised skin barriers and immune dysregulation following burn injuries [18]. This observation underscores the importance of considering the background testing frequency when comparing and interpreting the prevalence of infections in these patient populations.

The highest prevalence of bacteremia was documented the second week of ICU stay in both patient groups. Overall, the majority of identified pathogens was Gram-negative (61%). The pathogen spectrum revealed a predominance of *Enterobacter* spp., *S. aureus*, and *Klebsiella* spp. in burn patients and *Enterococcus* spp., *S. aureus*, and *P. aeruginosa* in the non-burn group. This distribution pattern of pathogens is largely consistent with the results documented in existing literature [19–21].

The prevalence of infections attributed to MDR bacteria in ICU environments is substantial and shows an ongoing global increase [22, 23]. In our study, the prevalence of MDR pathogens in 16.2% of identified pathogens, predominantly Gram-negative, is a notable concern. MDR resistance was mainly observed in the expected Gram-negative bacteria: *P. aeruginosa*, *K. pneumoniae*, and *A. baumannii*. The increased 60-day all-cause mortality observed in patients with MDR pathogens compared to those without bacteremia (41.7% versus 10.6%) accentuates the critical need for effective antimicrobial stewardship and infection control strategies in the ICU.

Our analysis revealed no significant difference in 60-day all-cause mortality between patients with or without burns. This suggests that severe burn trauma is associated with a high mortality, comparable to that of general ICU patients, which are typically older and have more comorbidities. Furthermore, due to the extensive damage and complexity of treatment required for burn injuries, including higher risk of (local/wound) infections, ongoing wound care, and multiple surgical interventions, the length of stay was also significantly longer in patients with burn trauma. Although infections are considered to be the primary cause of mortality in patients with severe thermal trauma, previous literature has reported inconsistent findings regarding the impact of bacteremia on mortality [6, 18, 24]. Our mortality analysis revealed no significant differences between patients with and without bacteremia, irrespective of burn trauma. This finding is at odds with the general expectation of higher mortality in patients with bacteremia. However, the absence of a significant mortality difference in patients with versus without bacteremia was only evident for susceptible pathogens and may be attributed to effective clinical management and timely intervention after identification of pathogens and subsequent antimicrobial susceptibility testing.

Strengths of our study include a direct comparison between intensive care patients with versus without burns within the same intensive care unit, providing comparable treatment practices. Furthermore, over 300 patients admitted to the ICU due to burn injuries represent a considerable sample size. Limitations of our study include its retrospective nature and single-center design, which may limit the generalizability of our findings. In addition, since most reasons for ICU admission include a combination of several diagnoses, we were unable to identify the precise reason for admission in non-burn patients. Furthermore, we were unable to distinguish between primary and definite catheter-related bloodstream or secondary nosocomial bloodstream infections. Finally, the non-burn group comprised a diverse population with various underlying medical and surgical conditions, potentially affecting comparability. However, it represented a well-balanced general ICU population, serving effectively as a suitable control group.

## Conclusion

In conclusion, the prevalence of bacteremia was similar between patients with burn injuries and a general ICU population. The frequency of multidrug resistance was high, especially in Gram-negative pathogens, and was associated with an increased 60-day all-cause mortality. There was no significant difference in 60-day all-cause mortality between patients with burns versus those without burns.

## Data Availability

Anonymized data and materials may be made available upon reasonable request.
